# An Account of Diseases in an Eastern District of London, from April 20 to May 20, 1806

**Published:** 1806-06

**Authors:** 


### *An Account of Diseases in an Eastern District of London, from April 20 to May 20, 1806.*

| | |
|---|---|
| ACUTE DISEASES. | |
| Typhus - - - | 2 |
| Peripneumonia - - | 6 |
| Rubeola - - - | 1 |
| Ophthalmia - - - | 7 |
| Rheumatismus Acutus | 3 |
| CHRONIC DISEASES. | |
| Tussis - - - | 20 |

Dyspnœa

| | | | |
|---|---|---|---|
| Dyspnœa - - - | 14 | Dysenteria - - | 2 |
| Tussis cum Dyspnœa | 11 | Hypochondriasis - | 4 |
| Phthisis Pulmonalis - | 3 | Rheumatismus Chronicus | 20 |
| Hæmoptysis - - | 3 | PUERPERAL DISEASES. | |
| Ascites - - - | 2 | Menorrhagia Lochialis | 5 |
| Anasarca - - - | 4 | Ephemera - - | 10 |
| Hydrothorax - - | 1 | Mastodynia - - | 3 |
| Cephalalgia - - | 4 | INFANTILE DISEASES. | |
| Amenorrhœa - - | 3 | Aphthæ - - - | 3 |
| Chlorosis - - - | 7 | Herpes - - - | 4 |
| Fluor Albus - - - | 9 | Tinea - - - | 3 |
| Hæmorrhois - - | 4 | Vermes - - - | 2 |
| Diarrhœa - - - | 3 | Dentition - - - | 2 |

The north and north-easterly wind has lately prevailed in an unusual degree. During a period of many weeks it has continued in this direction with the exception only of a very few days. The sudden alternation of heat and cold has rendered it very difficult to adjust our clothing to the temperature of the air. During the periods when the sky has been free from clouds, and the sun has shone out clearly, even the crossing from one side of a street in the metropolis to the other, has been the changing of a warm for a cold climate, or the reverse. To these circumstances may be attributed the long continuance of those diseases which prevail chiefly in the months of winter, and which usually disappear as the spring advances.

Coughs and catarrhs, and in some instances the more alarming and fatal diseases of the chest, prevail in a considerable degree even to the present time. Ophthalmia has also occurred in a number of instances, and in some has been tedious and obstinate. When this complaint has affected the patient during the prevalence of a high wind, it has been common to refer it to the dust being blown into the eyes; but a cold wind is sufficient to produce this effect independently of this circumstance. The number of patients affected by rheumatism either of the acute or chronic species has been considerable, and, probably owing to the circumstances already stated, this disease has been tedious and difficult to cure.

